# Supplementary material for: Months-long seismicity transients preceding the 2023 MW 7.8 Kahramanmaraş earthquake, Türkiye
Source: Nat Commun. 2023 Nov 28;14:7534. doi: 10.1038/s41467-023-42419-8 (PMC10684546; doi:10.1038/s41467-023-42419-8)
Supplement: Supplementary file 1 — Supplementary Information [file 41467_2023_42419_MOESM1_ESM.pdf]

Supplementary Information

Months-long seismicity transients preceding the 2023 M_W 7.8 Kahramanmaraş earthquake, Türkiye

Kwiatek, G.[1], Martínez-Garzón, P.[1], Becker, D.[1], Dresen., G.[1,2], Cotton., F.[1,2], Beroza, G. C.[3], Acarel, D.[4], Ergintav, S.[5] and Bohnhoff., M.[1,6]

[1] Helmholtz Centre Potsdam GFZ German Research Centre for Geosciences, Potsdam, Germany.

[2] Institute of Geosciences, University of Potsdam, Potsdam, Germany.

[3] Department of Geophysics, Stanford University, California, US.

[4] Institute of Earth and Marine Sciences, Gebze Technical University, Gebze-Kocaeli, Turkey.

[5] Department of Geodesy, Kandilli Observatory and Earthquake Research Institute, Boğaziçi University, Çengelköy-Istanbul, Turkey.

[6] Free University Berlin, Institute of Geological Sciences, Berlin, Germany.

Supplementary Data

File: **enhanced_event_catalog.xlsx:**
Enhanced seismic catalog starting on January 1st 2023 up to the 2023 M_W 7.8 Kahramanmaraş earthquake containing 1055 seismic events successfully located using the single event method NonLinLoc. The catalog contains the following fields: id longitude latitude depth [km] date[yyyymmddTHHMMSS] Magnitude[ML].

Supplementary Figures

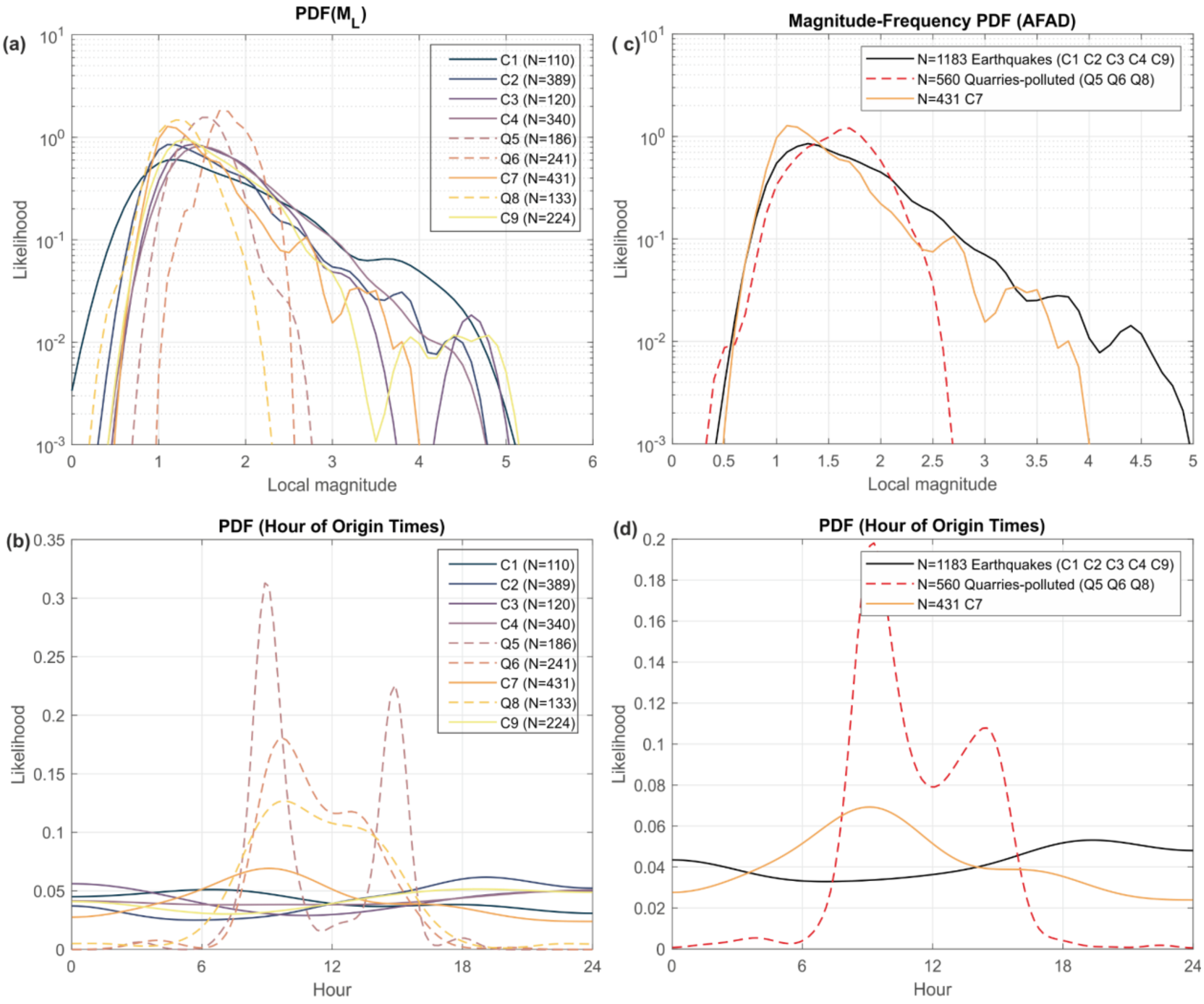

Supplementary Figure 1: **Identification of seismicity clusters and quarry blasts.** Statistics for seismicity clusters and quarry blasts are represented with solid and dashed lines, respectively. (a) Probability density function (PDF) of the earthquake magnitudes from each cluster calculated using the non-parametric kernel density approach. (b) PDF of the hours of event origin time calculated as in (a). In (a) and (b), colour is encoded with cluster number. (c, d) Same as (a, b) but stacking the PDFs from the earthquake and quarry blast clusters. C7 displaying ambiguous features is shown independently.

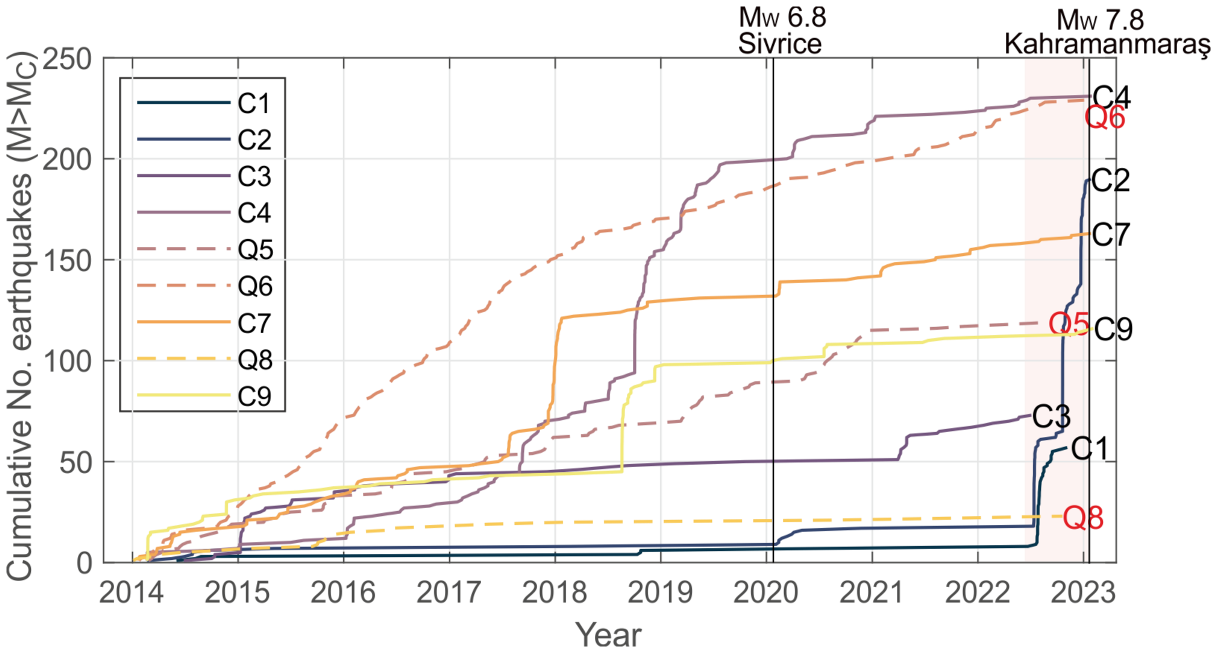

Supplementary Figure 2: Cumulative seismicity for the identified clusters. Acceleration of seismicity 8 months before the M_W 7.8 earthquake is visible for clusters C1 and C2. Clusters associated with quarry blasts (Q5, Q6, Q8) are plotted with dashed lines and red fonts. Color coding is the same as inFig. 2.

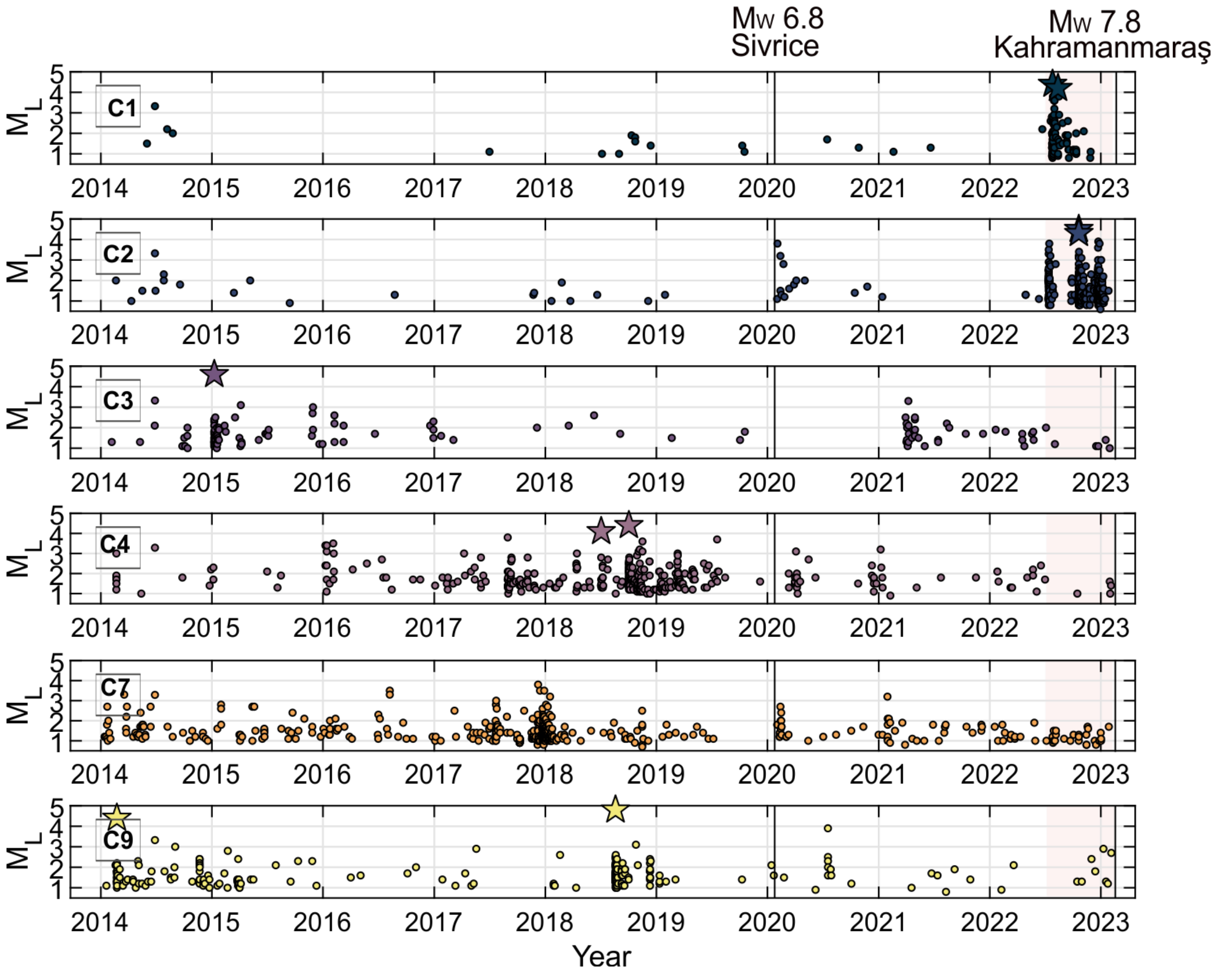

Supplementary Figure 3: **Seismicity in the analyzed seismicity clusters**. Local magnitudes and origin times from AFAD catalog[49] are shown for the time period 2014-2023. Events with M_L>4.0 are highlighted with stars.

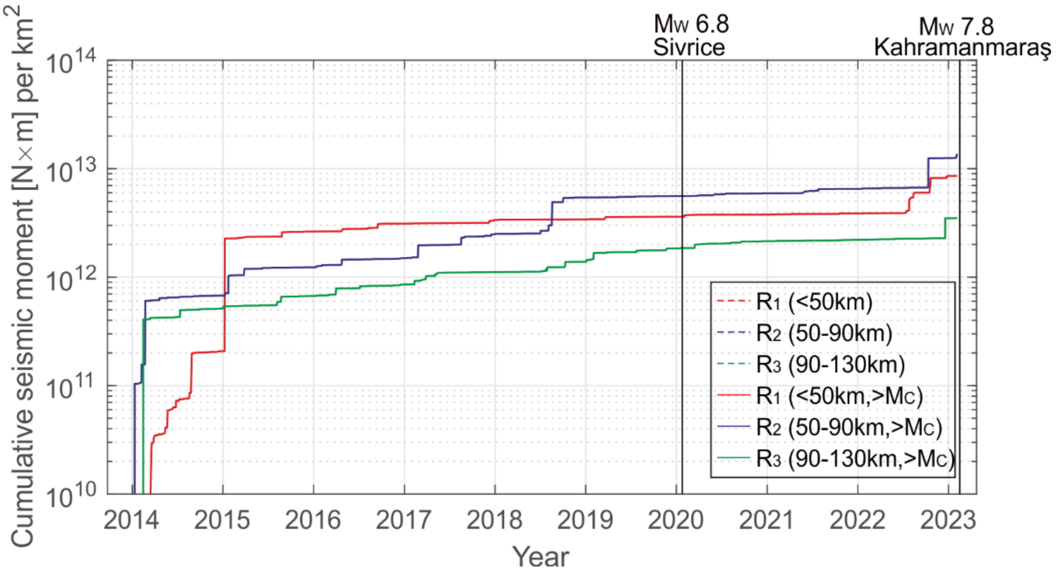

Supplementary Figure 4: Cumulative seismic moment release per km^2 in the three zones with different radius surrounding the epicentre of the M_W 7.8 earthquake. Dashed and solid lines represent the seismic events included in the entire catalogue and only above M_C, respectively (note the lines for complete catalogues strongly overlap with that from full catalogues, thus they are not visible).

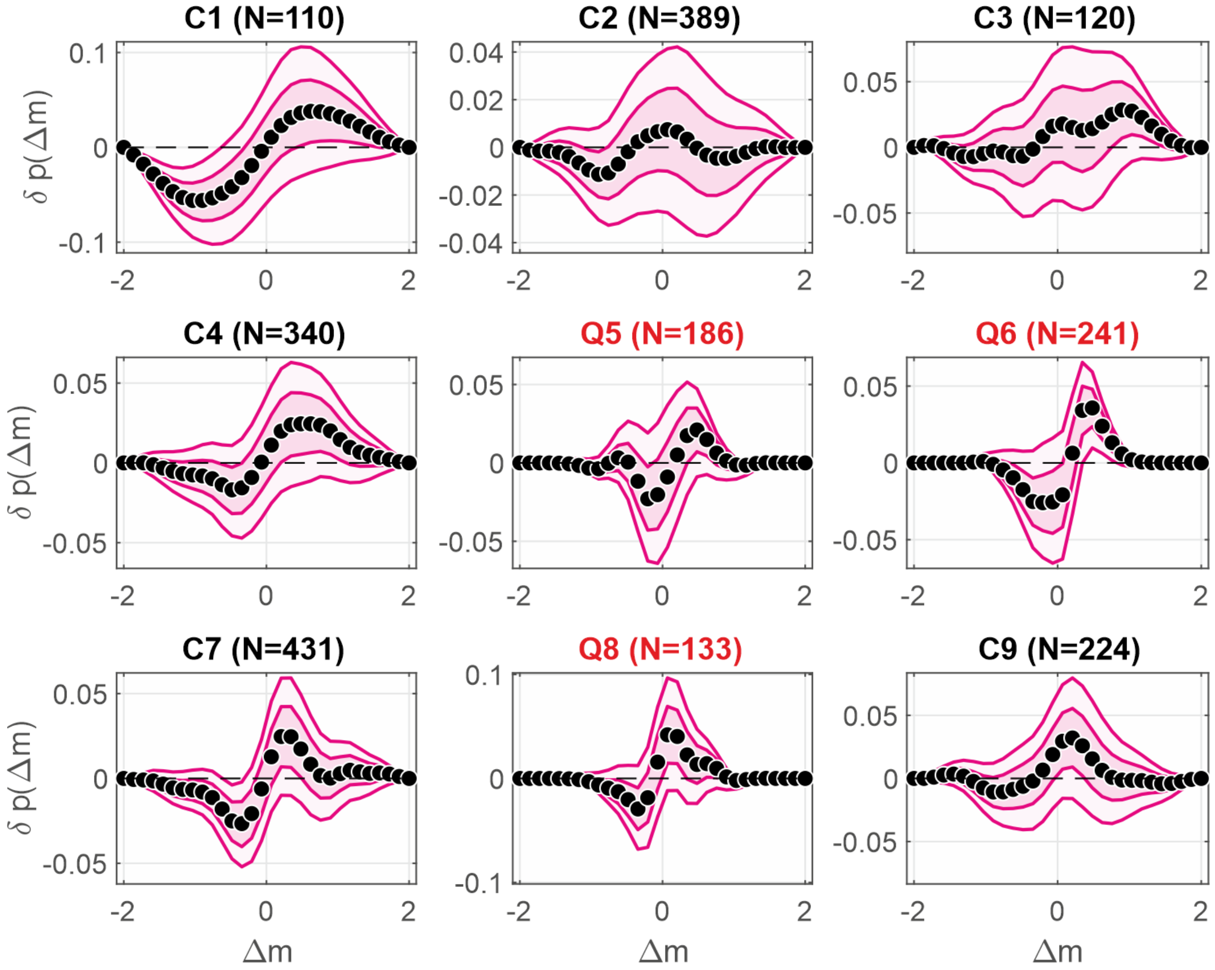

Supplementary Figure 5: **Magnitude correlations for identified clusters of seismicity and quarry blasts**. Differences in the probability to observe a magnitude difference $M_{i+1} - M_i < \Delta m$ between a selected subset of the catalog containing N earthquakes and its randomized versions which do not exhibit magnitude correlations (eq. 5) for clusters containing seismic activity C1-C4, C6, C7, C9 (title in black) and quarry blasts Q5, Q6, and Q8 (title in red). Dark and light magenta areas correspond to 68% and 95% confidence intervals, respectively. Statistically significant correlations in magnitudes correspond to significant deviations of the confidence intervals from zero baseline. Cluster C1 shows strong signatures of magnitude correlations.

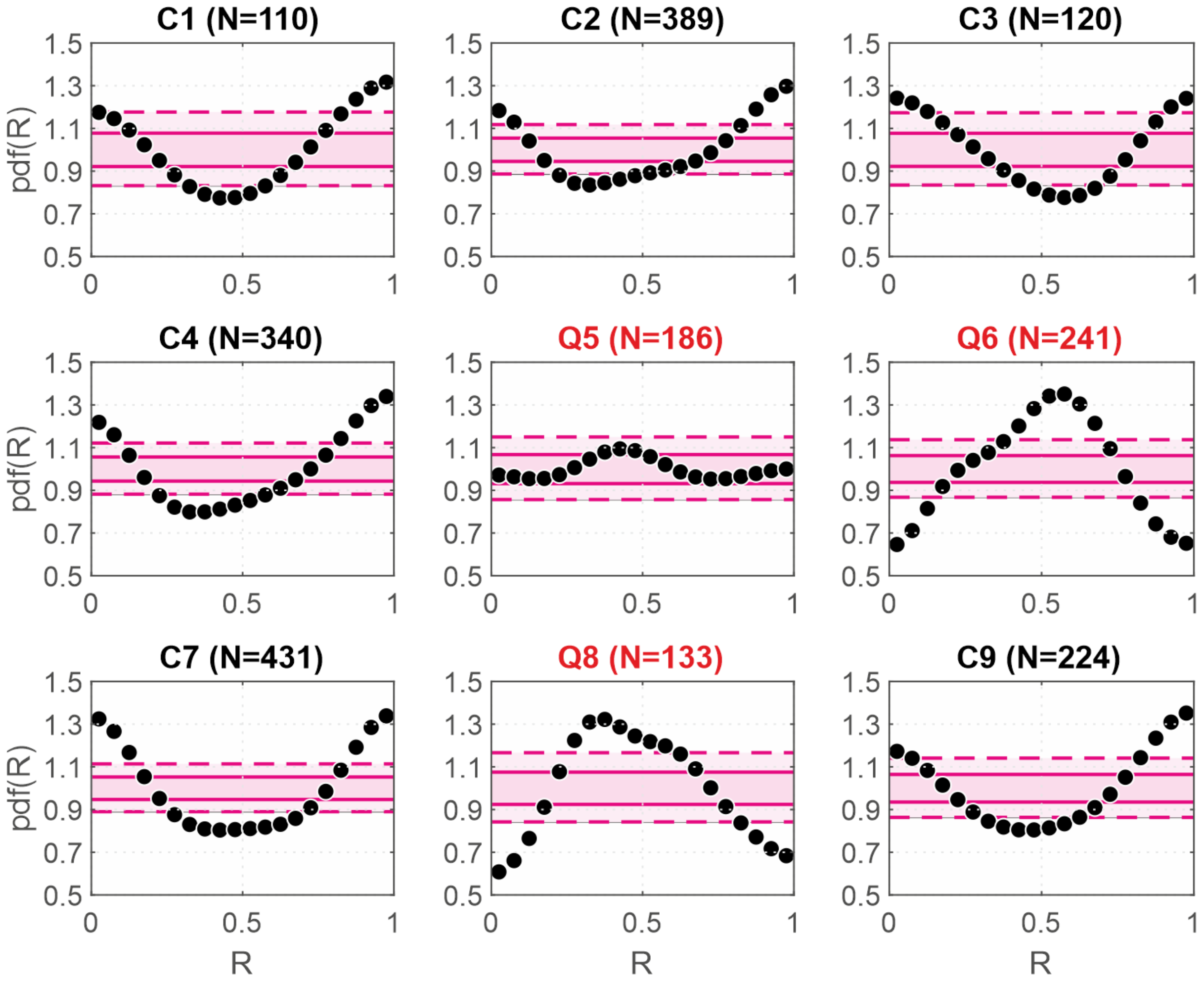

Supplementary Figure 6: **Interevent time ratios for identified clusters of earthquakes and quarry blasts**. Probability distribution functions of interevent times, $p(R)$ for clusters containing seismic activity C1-C4, C6, C7, -C9 (title in black) and quarry blasts Q5, Q6, and Q8 (title in red).. Dark and light magenta areas correspond to 68% and 95% confidence intervals expected from events randomly distributed in time (i.e. following a Poissonian process) assuming the same number of events as that in the selected cluster. Clusters containing seismic activity show statistically significant (anti-)clustering of seismicity in time. The distribution of inter-event times in Q6 and Q8 is degenerated suggesting repetitive events occurring in the cluster, here serving as an additional indicator for the presence of quarry blasts. The distribution of inter-event times in Q5 does not stray significantly from that expected from random distribution of earthquakes in time.

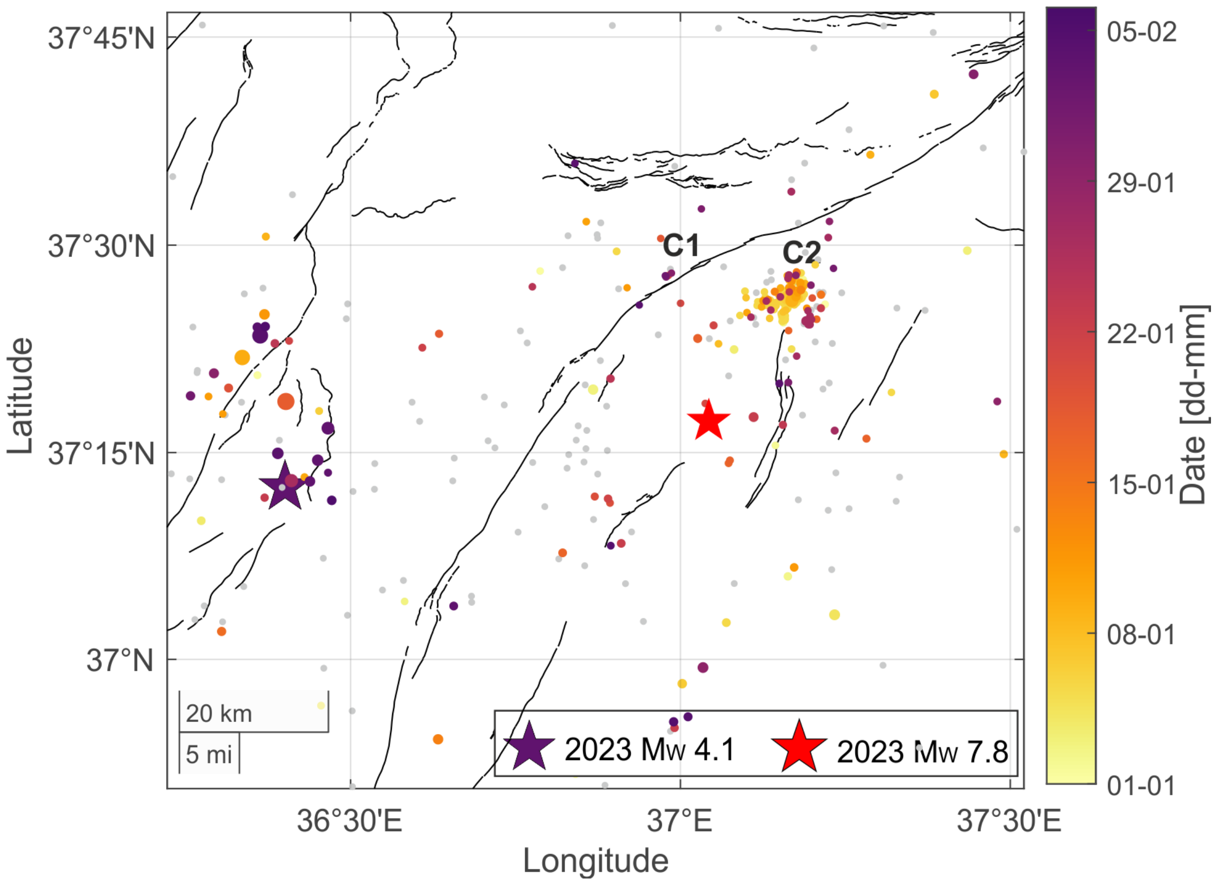

Supplementary Figure 7: Seismicity from the enhanced single event NLLoc catalogue around the nucleation area of the M_W7.8 Kahramanmaraş earthquake. The seismicity covers the time period from January 1st, 2023 until the origin time of the M_W7.8 earthquake. Coloured dots indicate single events NLLoc locations with major error ellipsoid half-axis < 10km and grey dots indicate all other events.

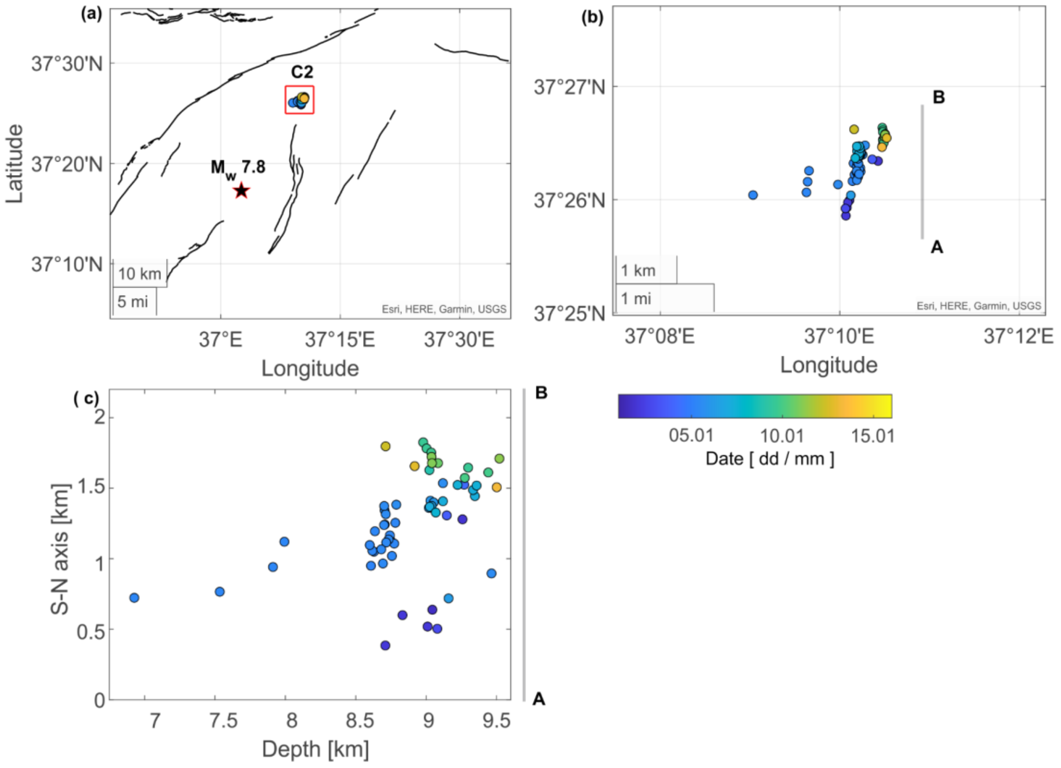

Supplementary Figure 8: Double-difference hypoDD relocation of the activity in cluster C2 from the enhanced seismicity catalogue. (a) Location of activity with respect to the mapped fault structures in the vicinity of the nucleation point of the M_W7.8 mainshock. (b) Close-up of the activity of cluster C2 from 01.01.2023-16.01.2023. Map boundaries are indicated by a red rectangle in (a). (c) Depth section of the C2 seismicity shown in (b).

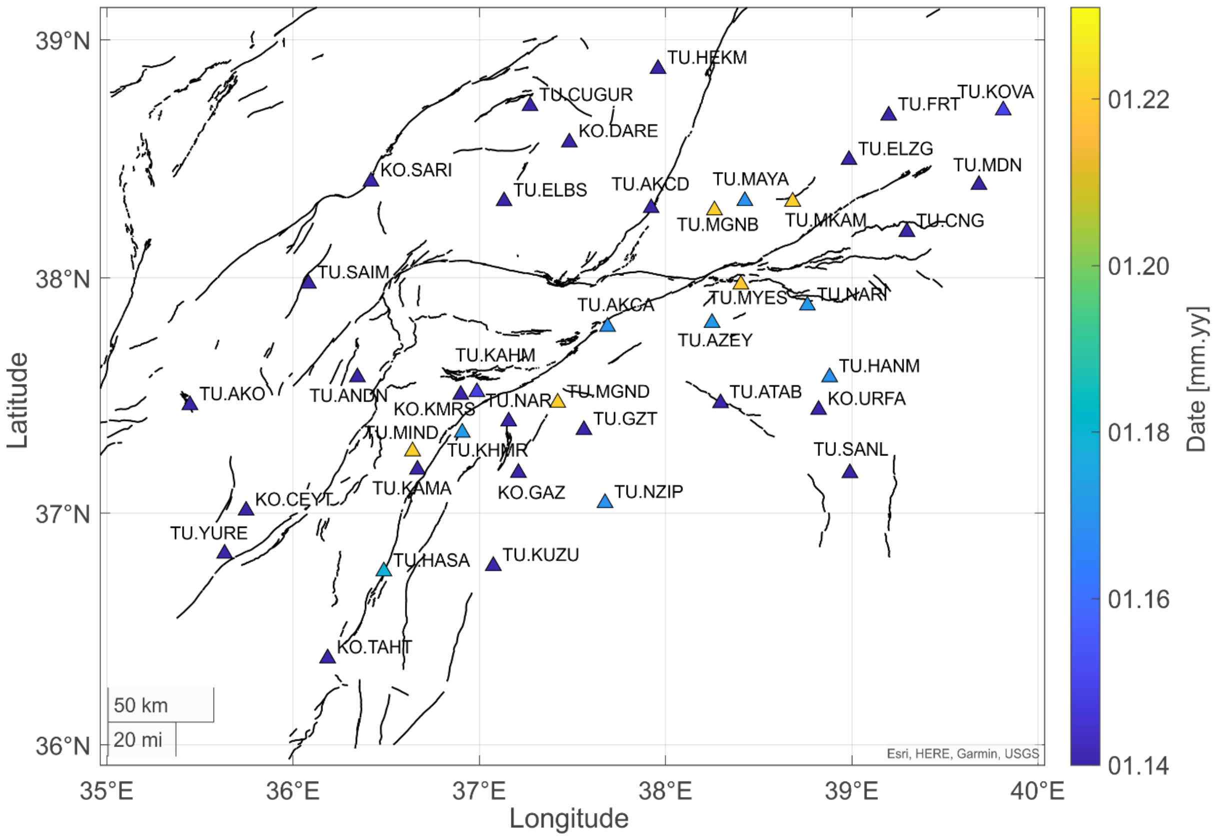

Supplementary Figure 9: **Map of AFAD stations colour-coded with the start time of operation**.
