# Supplementary material for: Months-long seismicity transients preceding the 2023 MW 7.8 Kahramanmaraş earthquake, Türkiye
Source: Nat Commun. 2023 Nov 28;14:7534. doi: 10.1038/s41467-023-42419-8 (PMC10684546; doi:10.1038/s41467-023-42419-8)
Supplement: Supplementary file 3 — Description of Additional Supplementary Files [file 41467_2023_42419_MOESM3_ESM.docx]

**Description of Additional Supplementary Files**

**Supplementary Dataset 1:** The excel file “enhanced_event_catalog.xls“ contains the data from the enhanced seismic catalog. It starts on January 1st, 2023, up to the 2023 MW 7.8 Kahramanmaraş earthquake and it contains 1055 seismic events successfully located using the single event method NonLinLoc. The catalog contains the following fields: id longitude latitude depth [km] date[yyyymmddTHHMMSS] Magnitude[ML].
